# The Clinical Impact of Access Site Selection for Successful Thrombolysis and Intervention in Acute Critical Lower Limb Ischaemia (RAD-ALI Registry)

**DOI:** 10.3390/life14060666

**Published:** 2024-05-23

**Authors:** Adam Csavajda, Karoly Toth, Nandor Kovacs, Szilard Rona, Zoltan Vamosi, Balazs Berta, Flora Zsofia Kulcsar, Olivier F. Bertrand, Istvan Hizoh, Zoltan Ruzsa

**Affiliations:** 1 Department of Invasive Cardiology, Bacs-Kiskun County Hospital, Teaching Hospital of the Albert Szent-Györgyi Medical School, University of Szeged, Nyiri Street 38, 6000 Kecskemet, Hungary; tothk@kmk.hu (K.T.); nandikov@gmail.com (N.K.); ronasz@kmk.hu (S.R.); vamosiz@kmk.hu (Z.V.); bberta9@gmail.com (B.B.); 2 Heart and Vascular Centre, Semmelweis University, Varosmajor Street 68, 1122 Budapest, Hungary; kulcsar.flora@med.semmelweis-univ.hu (F.Z.K.); ihizoh@web.de (I.H.); 3 Quebec Heart-Lung Institute, Laval University, Quebec City, QC G1V 4G5, Canada; olivier.bertrand@fmed.ulaval.ca; 4 Department of Internal Medicine, Invasive Cardiology, Albert Szent-Györgyi Medical School, University of Szeged, Semmelweis Street 8, 6725 Szeged, Hungary; zruzsa25@gmail.com

**Keywords:** acute limb ischaemia, angioplasty, endovascular treatment, local thrombolytic therapy, peripheral artery disease

## Abstract

**Background:** Acute limb ischaemia (ALI) is of great clinical importance due to its consequent serious complications and high comorbidity and mortality rates. The purpose of this study was to compare the acute success and complication rates of CDT performed via transradial, transbrachial, and transfemoral access sites in patients with acute lower limb vascular occlusion and to investigate the 1-year outcomes of CDT and MT for ALI. **Methods:** Between 2008 and 2019, 84 consecutive patients with ALI were treated with CDT in a large community hospital. Data were collected and retrospectively analysed. The primary (“safety”) endpoints encompassed major adverse events (MAEs), major adverse limb events (MALEs), and the occurrence of complications related to the access site. Secondary (“efficacy”) endpoints included both technical and clinical achievements, treatment success, fluoroscopy time, radiation dose, procedure time, and the crossover rate to an alternative puncture site. **Results:** CDT was started with radial (*n* = 17), brachial (*n* = 9), or femoral (*n* = 58) access. CDT was technically successful in 74/84 patients (88%), but additional MT and angioplasty and/or stent implantation was necessary in 17 (20.2%) and 45 cases (53.6%), respectively. Clinical success was achieved in 74/84 cases (88%). The mortality rate at 1 year was 14.3%. The cumulative incidence of MAEs and MALEs at 12 months was 50% and 40.5%, respectively. After conducting multivariate analysis, history of Rutherford stage IIB (hazard ratio [HR], 3.64; 95% confidence interval [CI], 1.58–8.41; *p* = 0.0025), occlusion of the external iliac artery (HR, 27.52; 95% CI, 2.83–267.33; *p* = 0.0043), being a case of clinically unsuccessful thrombolysis (HR, 7.72; 95% CI, 2.48–23.10; *p* = 0.0004), and the presence of diabetes mellitus (HR, 2.18; 95% CI, 1.01–4.71; *p* = 0.047) were independent predictors of a high MAE mortality rate at 12 months. For MALEs, statistically significant differences were detected with the variables history of Rutherford stage IIB (HR, 4.30; 95% CI, 1.99–9.31; *p* = 0.0002) and external iliac artery occlusion (HR, 31.27; 95% CI, 3.47–282.23; *p* = 0.0022). **Conclusions:** Based on the short-term results of CDT, acute limb ischaemia can be successfully, safely, and effectively treated with catheter-directed thrombolytic therapy with radial, brachial, or femoral access. However, radial access is associated with fewer access site complications. A history of Rutherford stage IIB, occlusion of external iliac artery, unsuccessful thrombolysis, and the presence of diabetes mellitus were independently associated with an increased risk of MAEs. A history of Rutherford stage IIB and external iliac artery occlusion are independent predictors of MALEs.

## 1. Introduction

Acute lower limb ischaemia (ALI) is a sudden decrease in perfusion of the lower limb that entails a potential threat to the viability of the limb. Symptoms may manifest as pain at rest or, in more severe cases, as tissue damage in the form of ulcers and/or gangrene. Among the treatment options for endangered limbs are catheter-directed thrombolysis (CDT), mechanical thrombectomy (MT), and surgical revascularisation. ALI and its treatment carry the risk of complications such as ischaemia–reperfusion injury, compartment syndrome, systemic inflammatory response syndrome, multiple organ dysfunction syndrome, hyperkalaemia, or bleeding. Current advances in endovascular therapy enable prompt treatment of ALI; however, most treatment is performed via traditional femoral access sites. Alternatives to femoral access in ALI are the upper limb access sites of the radial and brachial arteries [1,2] or pedal access [3,4,5], but these access sites have many limitations in everyday clinical practice. 

Our aim was to investigate the rate of complications related to the access site and to evaluate predictors of long-term outcomes of CDT and MT in lower limb arteries.

## 2. Materials and Methods

Between 2008 and 2019, consecutive patients with ALI were treated with CDT in a large community hospital. We collected the data of these patients and performed a retrospective analysis. We enrolled only patients who, based on the decision of the vascular team, underwent CDT. Based on the artery used as the access site, patients were divided into the RA (radial artery; *n* = 17), BA (brachial artery; *n* = 9), and FA (femoral artery; *n* = 58) groups. The operator selected the access site and all procedures were performed by five skilled operators. 

Ad hoc informed consent for the procedure was obtained from all patients. The National Ethical Review Committee approved the study (reference number, BMEÜ/1639-1/2022/EKU) and all patients provided written informed consent prior to their inclusion in the study.

### 2.1. Procedural Endpoints

The primary endpoints of the study were “safety endpoints”: major adverse events (MAEs), major adverse limb events (MALEs), and occurrences of complications related to the access site. Secondary endpoints were “efficacy endpoints”: technical and clinical success, efficacy of the treatment, fluoroscopy time, radiation dose, procedure time, and the crossover rate to an alternative puncture site. 

### 2.2. Inclusion and Exclusion Criteria

Inclusion criteria: Patients with ALI classified as Rutherford stage I, IIA, or IIB; acute lower extremity vascular occlusion confirmed based on emergency diagnostic angiography; clinic attendance starting within 14 days; age > 18 years; and had signed the patient information sheet and consent form were included. 

Exclusion criteria: Patients were excluded if they had a non-viable lower limb (Rutherford stage III); did not sign the patient information sheet or the consent form; had haemodynamic instability; did not have significant vascular occlusion on diagnostic angiography; were not admitted to the clinic within 14 days; age < 18 years; or had inflammatory skin lesions at the planned penetration sites. 

### 2.3. Antithrombotic and Thrombolytic Regimen

CDT involved the use of a recombinant tissue plasminogen activator administered directly into the artery. It consisted of an initial dose of 10 mg followed by a maintenance dose of 1 mg/h. Intravenous sodium heparin was administered to prevent catheter thrombosis. The heparin was administered as an initial bolus of 60 IU/kg and a maintenance dose of 1000 IU/h, adjusted based on the activated partial thromboplastin time.

Following the administration of an initial dose of 300 mg aspirin and 300 mg clopidogrel, individuals who underwent stenting procedures were prescribed a dual antiplatelet therapy regimen consisting of 100 mg aspirin and 75 mg clopidogrel daily for 2 months. Conversely, patients who solely underwent balloon angioplasty were prescribed lifelong aspirin therapy.

### 2.4. Catheter Directed Thrombolysis 

**Figure 1:**
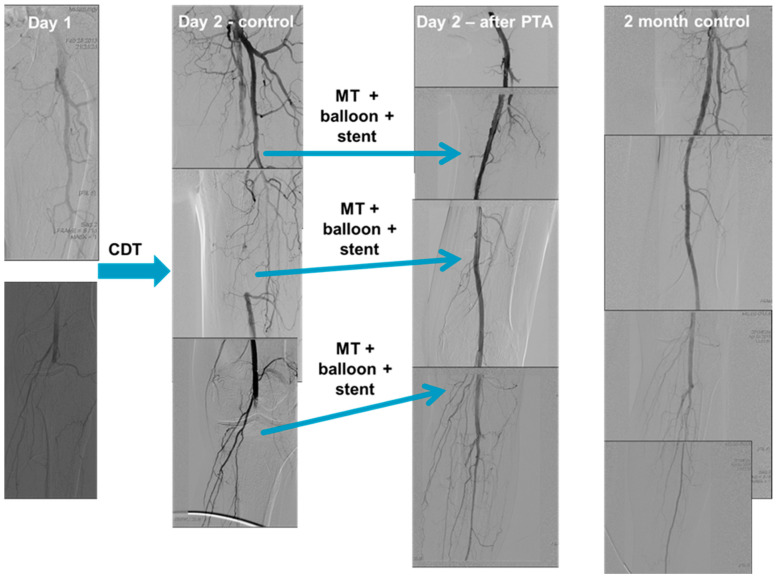
Catheter-directed thrombolysis (CDT) performed via access from a radial artery. Selective angiography performed using a radial approach (over a 5F pigtail catheter) shows a left common femoral artery occlusion without distal run-off. (Day 1) A guidewire transversal test, in which the lesion was passed with an 0.18-inch guidewire, was conducted over a multiport thrombolytic catheter. CDT was then initiated. On the first postoperative day, control angiography shows incomplete thrombus resolution and distal embolisation. (Day 2—control) Mechanical thrombectomy, additional balloon angioplasty, and stent implantation was performed in the left superficial femoral artery and in the left popliteal artery. Control angiography shows successful recanalisation with acceptable flow in the below the knee arteries. (Day 2—after PTA). Abbreviations: CDT—catheter-directed thrombolytic therapy; MT—mechanical thrombectomy; PTA—percutaneous transluminal angioplasty.

CDT was performed after selective angiography over a 5F pigtail catheter. The CDT was always initiated after a guidewire transversal test was conducted (the lesion was passed with a 0.18-inch guidewire) over a multiport thrombolytic catheter. If a CDT therapy was initiated, a control angiography was performed 24 h after local administration of a thrombolytic drug. If the thrombus continued to impede or completely block the flow despite the use of thrombolysis, additional thrombus aspiration was conducted. Similarly, if the lesion was significantly stenosed or dissected, additional balloon angioplasty or stent implantation was performed (Figure 1). The choice of treatment varied based on the location of the occlusion, the extent of clot formation, and the aetiology. 

In cases of thromboembolism, vascular surgery was the preferred treatment and the decision was reached after consultation among the members of the vascular team. In cases of atherothrombosis, CDT was the preferred treatment, and the vascular team also made this decision. Based on the above, only patients who underwent thrombolysis were included in our present study.

### 2.5. Definitions

#### 2.5.1. Major Adverse Event

An MAE was evaluated by considering a combination of outcomes, including death, myocardial infarction, stroke, major amputation of the lower limb, and the need for repeat revascularisation procedures of the target vessel by percutaneous transluminal angioplasty or by arterial bypass graft surgery during the follow-up period.

#### 2.5.2. Major Adverse Limb Event

A MALE was defined as either untreated loss of patency of the revascularisation, re-intervention on the revascularised segment, or major amputation (above or below the knee) of the revascularised limb. 

#### 2.5.3. Vascular Complication

Major vascular complications referred to a reduction in, or total loss of, the arterial pulse or the emergence of a pseudoaneurysm or arteriovenous fistula as identified during the patient’s follow-up examination. Minor vascular complications were characterised as haematomas that did not necessitate any specific intervention. These haematomas were limited to a size of 2 cm in diameter in the puncture areas of the radial or ulnar artery, or 5 cm in diameter in the puncture areas of the femoral or brachial arteries. A drop in the haemoglobin level of more than 3 g/dL was considered major bleeding, as was bleeding that required transfusion.

#### 2.5.4. Technical Success

The successful outcome of a technical procedure occurred when a percutaneous transluminal angioplasty led to a residual stenosis of less than 30% while ensuring satisfactory anterograde blood flow. A suboptimal result was identified by a slow flow and/or a residual stenosis of between 30% and 50% after percutaneous transluminal angioplasty. 

#### 2.5.5. Clinical Success

The primary measure of clinical success involved observing an enhancement of at least one clinical category within the Rutherford classification [6]. Primary patency referred to the condition where a treated lesion remained open and unobstructed over time, without requiring any additional medical procedures such as angioplasty, surgery, or amputation. Limb salvage was the successful prevention of major amputation, preserving the affected limb. We also evaluated the treatment as a success if the functionality of the limb was maintained in the first 7 days and no major amputation occurred. 

#### 2.5.6. Access Site Crossover

If technical difficulties arose in connection with the intervention performed through the primary penetration site, or if performing the intervention from this puncture point was not possible, the use of a ‘crossover’ site was deemed necessary. That is, the puncture area was switched to another puncture area.

### 2.6. Follow-Up

All patients underwent a physical examination immediately after the procedure and every day during hospitalisation. In the third, sixth, and twelfth months after the intervention, a detailed clinical follow-up examination was performed on all patients.

### 2.7. Statistical Analysis

The unordered chi-squared test with a simulated *p*-value (10^7^ replicates) was employed to evaluate categorical data. Pairwise comparisons were made using Fisher’s exact test adjusted according to the Benjamini–Hochberg method to account for multiple comparisons [7]. For ordered larger contingency tables, the ordered approximative general independence test (10^7^ resamples) was employed. All continuous parameters showed non-normal distribution; therefore, they were described using the median and interquartile range. The three treatment groups were evaluated using the approximative Kruskal–Wallis’s test (10^7^ resamples), using the Dunn’s test and the Holm adjustment as a post hoc test in cases of statistical significance. The null hypothesis was rejected if *p* was ≤0.025. All analyses were carried out with R version 4.3.1 (R Foundation for Statistical Computing, Vienna, Austria) using the additional packages coin 1.4-2, R companion 2.4.30, and Dunn test 1.3.5. All analyses were conducted on an intention-to-treat basis. Cox regression was performed using MedCalc version 22.016 (MedCalc Software Ltd., Ostend, Belgium) statistical software. *p* < 0.05 was considered statistically significant.

## 3. Results

Between 2008 and 2019, 84 consecutive patients fulfilling the inclusion criteria underwent CDT for ALI, and their data were analysed retrospectively. CDT was initiated using radial (RA group, *n* = 17), brachial (BA group, *n* = 9), and femoral (FA group, *n* = 58) approaches. The demographic and clinical data are summarised in Table 1. 

### 3.1. Angiographic and Procedural Data

The angiographic and procedural data are summarised in Table 2 and Table 3. CDT was technically successful in 74/84 patients (88%), but additional MT and angioplasty and/or stent implantation was necessary in 17 (20.2%) and 45 (53.6%) cases, respectively, to obtain good angiographic results. Clinical success was achieved in 74/84 cases (88%). Procedurally related factors were not statistically different in the subgroups (see Table 3) and hospitalisation time (15.9 ± 14.5 days vs. 7.7 ± 2.8 days vs. 11.5 ± 6 days) was also not statistically different among the three groups (*p* = ns). 

### 3.2. Procedural Complications and 1-Year Follow-Up

The procedural complications and long-term follow-up data are summarised in Table 3 and Table 4. The cumulative incidence of MAEs at 12 months was 50%. The major amputation rate was 22.6% and the mortality rate 21.5% (regarding patients who have undergone major amputation). Among the major amputations performed, a significant proportion (73,7%) were femoral amputations. Four patients were identified as having stage IIA according to the Rutherford classification, while 10 had stage IIB. Crural amputations represented 26.3% of the overall number of amputations. Within this subset, four patients were classified as having stage IIA according to the Rutherford classification and one as having stage IIB. The overall rate of stroke was 9.5%: five (5.9%) cases of ischaemic stroke and three (3.5%) of haemorrhagic stroke, with a 50% mortality rate. The overall rate of major vascular complication was 9.5% (0%, 11.1%, and 12.1% in the RA, BA, and FA groups, respectively), with a 25% mortality rate.

### 3.3. MAE and MALE Predictors

The adjusted variables used in the Cox regression model used to investigate MAEs and MALEs, which were considered together as the reference model, were entry site, Rutherford stage, target vessel, clinical success, additional procedure, and diabetes mellitus. In all cases, data from the 12-month follow-up period were taken into account during the analysis, and *p* < 0.05 was considered statistically significant.

Among the adjusted variables examined for MAEs, statistically significant differences were observed for Rutherford stage IIB (HR, 3.64; 95% CI, 1.58–8.41; *p* = 0.0025), occlusion at the level of the external iliac artery (HR, 27.52; 95% CI, 2.83–267.33; *p* = 0.0043), cases of clinically unsuccessful thrombolysis (HR, 7.72; 95% CI, 2.48–23.10; *p* = 0.0004), the presence of diabetes mellitus (HR, 2.18; 95% CI, 1.01–4.71; *p* = 0.047), and cases of radial penetration (HR, 0.27; 95% CI, 0.07–0.96; *p* = 0.0429; Table 5). 

In the case of MALEs, statistically significant differences were detected for the variables Rutherford stage IIB (HR, 4.30; 95% CI, 1.99–9.31; *p* = 0.0002) and external iliac artery occlusion (HR, 31.27; 95% CI, 3.47–282.23; *p* = 0.0022; Table 6).

## 4. Discussion

This is the first study to compare the use of femoral, brachial, and radial access for the treatment of ALI. We demonstrated several important findings: (1) based on the short-term results, CDT was a safe and effective method to treat ALI; however, additional intervention was necessary in 73.8% of cases; (2) access site complications were very rare in the RA group; (3) long-term MAEs were frequent, despite CDT being successful; and (4) significant independent predictors of long-term MAEs and MALEs were identified.

ALI treatment encompasses various methods, including surgical interventions such as thromboembolectomy and bypass surgery, and endovascular techniques such as CDT, percutaneous thrombus aspiration, mechanical thrombectomy, angioplasty, and stent placement. A hybrid treatment approach that combines both surgical and endovascular therapies is also available.

According to the 2016 American Heart Association/American College of Cardiology (AHA/ACC) consensus guidelines for managing peripheral arterial disease, thrombectomy is indicated as ‘IIa’ in patients with ALI caused by embolism and whose limbs can still be saved [8]. Two major studies were published in the 1990s that proved the noninferiority of endovascular therapy over surgical thrombectomy. These were the Surgery or Thrombolysis in Lower Extremity Ischemia (STILE) and Thrombolysis Or Peripheral Artery Surgery (TOPAS) trials, which randomly assigned patients to arteriography and lysis therapy with urokinase versus arteriography and embolectomy or urgent bypass [9,10]. The TOPAS study showed that local thrombolysis with urokinase is associated with a higher rate of bleeding complications, but simultaneously reduces the need for open surgical interventions without significantly increasing the risk of amputation or death [10]. The data interpreted in the studies are reliable and the test results are well founded; however, in both studies, the majority of patients had stage I and IIA limb ischaemia, while stage IIB and III ischaemia occurred in less than 25% of patients. 

The primary goal of CDT is to restore anterograde flow, clear the outflow tract of thrombus, minimise the level of amputation, and allow for further peripheral intervention without distal embolisation developing. MT can be used to supplement CDT. MT utilises the fusion of mechanical energy and thrombolytic infusion to actively disintegrate and dissolve the thrombus. MT enables quicker dissolution of clots compared to CDT, although it may also entail an increased risk of bleeding and the occurrence of distal embolisation [11].

In the past, CDT was associated with a longer treatment time (infusion of the thrombolytic), often wasting valuable days to achieve any degree of recanalisation. Nowadays, the time to perfusion can be significantly shortened using thrombolytic protocols, which have been accelerated by technical advances. In our study, we verified and confirmed that currently, CDT is an effective therapeutic intervention in ALI, as local thrombolytic therapy was technically and clinically successful in 88% of patients. 

Advances in endovascular techniques are increasingly enabling ALI to be treated; however, most treatments are still performed via the traditional femoral access. Alternative entry points, such as brachial or radial artery punctures, can also be used [12,13]. The main disadvantages of transfemoral access are the high rate of vascular complications related to the puncture and the longer duration of hospitalisation [14]. Ultrasound-guided puncture might result in fewer vascular complications and avoidance of multiple punctures. 

Compared to femoral penetration, using radial penetration offers the main advantages of a much lower rate of bleeding complications, greater comfort of the patient, enabling of rapid mobilisation, and shorter hospital stays [13,14]. Despite its many advantages, the anatomical and technical limitations of the radial approach have prevented it from being widely used and accepted as a penetration approach. The most important disadvantage of this approach is the difficulty in delivering devices to the lesion to be treated and the high crossover rate to the femoral access site [15]. Owing to technological improvements, the use of transradial access for endovascular interventions has become much more feasible [16]. One of the main advantages of radial penetration was confirmed by our present study: the overall rate of major vascular complications was 9.5% (0%, 11.1%, and 12.1% in the RA, BA, and FA groups, respectively), with a 25% mortality rate.

### 4.1. Acute and Long-Term Results of CDT

Our technical success rate of 88% is similar to the reported rates of previous studies investigating ALI and thrombolysis [10,17]. CDT shows high angiographic success in treating ALI; however, long-term outcomes are relatively poor, with a relatively high risk of MAEs (the cumulative incidence of MAEs at 12 months was 50%, with a 22.6% rate of major amputation). Potential confounding factors such as age and comorbidities may underlie the outcomes of CDT. In the present study, the comorbidities among the patient population and potential risk factors were clearly visible: the majority of patients had hypertension and were previous or active smokers, while approximately one third of the patients had diabetes or previous PTA.

Using multiple regression analysis enabled those factors that significantly increased the rate of MAEs during long-term follow-up to be determined. These risk factors were primarily a high initial Rutherford stage, a high degree of occlusion, diabetes mellitus present as a comorbidity, or failure of thrombolysis. Regarding the occurrence of MAEs, patients who underwent the intervention from a radial penetration site had a significant advantage compared to those who underwent the intervention, for example, from a femoral penetration site. Regarding MALEs, patients with Rutherford stage IIB and external iliac artery occlusion who underwent treatment fared worse than did those with Rutherford stage IIA or, for example, patients with superficial femoral artery occlusion.

### 4.2. Complications of CDT 

Intracranial bleeding is the most serious, and often fatal, complication of CDT, despite therapy being initiated immediately. An urgent computed tomography scan of the skull, the immediate cessation of lysis, and the initiation of maximum supportive therapy are necessary if the patient’s neurological status is to be changed. However, even with the greatest care and maximum supportive therapy, the prognosis is very poor.

A published Cochrane meta-analysis assessed five trials with 1283 randomised patients comparing thrombolysis and surgery. The authors concluded that no significant difference in limb salvage or death at 30 days, 6 months, or 1 year existed between initial surgery and thrombolysis. However, major bleeding, stroke, and distal embolisation were more likely to be associated with thrombolysis than with thrombectomy [18]. This fact was confirmed by our present study, as the overall rate of stroke was 9.5% (five [5.9%] cases of ischaemic and three [3.5%] of haemorrhagic stroke), with a 50% mortality rate.

Another dreaded adverse event is major access site complications, which can increase the risk of mortality and the length of hospital stay and medical costs [19]. These complications comprise pseudoaneurysms or arteriovenous fistulae at the puncture site, or retroperitoneal haemorrhage, which requires immediate surgical intervention. The severity of these complications is confirmed by the results of the present study: the overall rate of major vascular complications was 9.5%, with a 25% intrahospital mortality rate. 

Based on the above, one can conclude that close monitoring and physical examination of the patient is essential after the interventions have been performed. 

### 4.3. Study Limitation

The primary limitation of the study was the nonrandomised, retrospective design. The small and unequal number of patients in each subgroup made extrapolation of their result over the entire population of ALI patients difficult.

## 5. Conclusions

According to the initial outcomes, acute limb ischaemia can be treated successfully and safely using radial, brachial, or femoral access sites; however, radial access is associated with fewer access site complications. Good patient outcomes depend on how quickly and effectively the arterial blood flow can be restored to the limb. Patients with greater ischaemic tissue damage (e.g., acute aortic occlusion) have much worse outcomes than those with segmental arterial occlusion have. Concerning long-term outcomes, MAEs are frequent despite CDT being conducted successfully. The reason for this could be the significant comorbidity of the patient population, the presence of a chronic component that frequently accompanies the acute clinic, and an initial high Rutherford stage.

## Figures and Tables

**Table 1:** Demographic and clinical data.

Variable	RA Group (*n* = 17)	BA Group (*n* = 9)	FA Group (*n* = 58)	*p* Value Overall	*p* Value RA vs. BA Groups	*p* Value RA vs. FA Groups	*p* Value BA vs. FA Groups
Age, median (IQR), years	67.0 (59.0–69.0)	60.0 (57.0–63.0)	64 (55.3–71.0)	0.4558	NA	NA	NA
Female	5 (29.4%)	2 (22.2%)	12 (20.7%)	0.8428	NA	NA	NA
BMI, median (IQR), kg/m^2^	29.8 (23.3–30.9)	23.1 (21.5–25.3)	25.3 (22.5–29.4)	0.1653	NA	NA	NA
Hypertension	16 (94.1%)	8 (88.9%)	48 (82.8%)	0.6326	NA	NA	NA
Current smoker	12 (70.6%)	7 (77.8%)	43 (74.1%)	1.0	NA	NA	NA
Diabetes mellitus	4 (23.5%)	3 (33.3%)	14 (24.1%)	0.8529	NA	NA	NA
CAD	2 (11.8%)	2 (22.2%)	9 (15.5%)	0.7936	NA	NA	NA
Previous PTA	4 (23.5%)	3 (33.3%)	18 (31.0%)	0.8683	NA	NA	NA
Chronic renal failure	1 (5.9%)	2 (22.2%)	7 (12.1%)	0.5044	NA	NA	NA
COPD	2 (11.8%)	5 (55.6%)	9 (15.5%)	0.0172 *	0.0424 *	1.0	0.0424 *
Clinical presentation				0.5966	NA	NA	NA
Rutherford stage I	0 (0.0%)	0 (0.0%)	0 (0.0%				
Rutherford stage IIA	13 (76.5%)	6 (66.7%)	47 (81.0%)				
Rutherford stage IIB	4 (23.5%)	3 (33.3%)	11 (19.0%)				
Rutherford stage III	0 (0.0%)	0 (0.0%)	0 (0.0%)				

Abbreviations: BA—brachial artery; BMI—body mass index; CAD—coronary artery disease; COPD—chronic obstructive pulmonary disease; FA—femoral artery; IQR—interquartile range; NA—not assessed; RA—radial artery; PTA—percutaneous transluminal angioplasty. Categorical outcomes: chi-squared test with simulated *p*-value (10^7^ replicates); post hoc test: Fisher’s exact test adjustment according to Benjamini–Hochberg method. For ordered larger contingency tables, the ordered approximative general independence test (10^7^ resamples) was used. Continuous outcomes: approximative Kruskal–Wallis’s test with 10^7^ resamples; post hoc test: Dunn’s test and the Holm adjustment; alpha = 0.05; reject H_0_ if *p* ≤ alpha/2. * Statistically significant.

**Table 2:** Angiographic data.

Variable	RA Group (*n* = 17)	BA Group (*n* = 9)	FA Group (*n* = 58)	*p* Value Overall	*p* Value RA vs. BA Groups	*p* Value RA vs. FA Groups	*p* Value BA vs. FA Groups
**Superficial femoral artery**							
Diameter stenosis, %	ND	ND	ND				
Lesion length, mm	ND	ND	ND				
Reference diameter, mm	ND	ND	ND				
**Popliteal artery**							
Diameter stenosis, %	ND	ND	ND				
Lesion length, mm	ND	ND	ND				
Reference diameter, mm	ND	ND	ND				
**Lesion type**				0.6932	NA	NA	NA
TASC A	0 (0.0%)	0 (0.0%)	0 (0.0%				
TASC B	3 (17.6%)	1 (11.1%)	4 (6.9%)				
TASC C	0 (0.0%)	1 (11.1%)	5 (8.6%)				
TASC D	14 (82.4%)	7 (77.8%)	49 (84.5%)				
CTO	1 (5.9%)	0 (%)	1 (1.7%)	0.5256	NA	NA	NA

Abbreviations: BA—brachial artery; CTO—chronic total occlusion; FA—femoral artery; NA—not assessed; ND—not determined; RA—radial artery; TASC—Trans-Atlantic Inter-Society Consensus. Categorical outcomes: chi-squared test with simulated *p*-value (10^7^ replicates); post hoc test: Fisher’s exact test adjustment according to Benjamini–Hochberg method. For ordered larger contingency tables, the ordered approximative general independence test (10^7^ resamples) was used. Continuous outcomes: approximative Kruskal–Wallis’s test with 10^7^ resamples; post hoc test: Dunn’s test and the Holm adjustment; alpha = 0.05; reject H_0_ if *p* ≤ alpha/2.

**Table 3:** Results.

Outcomes	RA Group (*n* = 17)	BA Group (*n* = 9)	FA Group (*n* = 58)	*p* Value Overall	*p* Value RA vs. BA Groups	*p* Value RA vs. FA Groups	*p* Value BA vs. FA Groups
Procedural success	ND	ND	ND	NA	NA	NA	NA
Clinical success	14 (82.4%)	8 (88.9%)	52 (89.7%)	0.8666	NA	NA	NA
Access site complications	0 (0.0%)	3 (33.3%)	18 (31.0%)	0.0254 *	0.0485 *	0.0235 *	1.0
Major adverse events at 12 months	7 (41.2%)	6 (66.7%)	29 (50.0%)	0.4879	NA	NA	NA
Crossover	8 (47.1%)	5 (55.6%)	50 (86.2%)	0.0021 *	1.0	0.0054 *	0.0708
Additional thrombectomy	3 (17.6%)	2 (22.2%)	12 (20.7%)	1.0	NA	NA	NA
Additional angioplasty/stent	8 (47.1%)	5 (55.6%)	32 (55.2%)	0.8883	NA	NA	NA
Median procedural time(IQR), minutes	40 (25.0–57.5)	82.5 (76.3–91.3)	45.0 (35.0–58.8)	0.0218 *	0.0135 *	0.3229	0.0076 *
Median fluoroscopy time(IQR), minutes	10.0 (6.4–19.5)	23.8 (19.7–28.0)	12.8 (8.4–18.4)	0.1111	NA	NA	NA
Median radiation dose(IQR), dyne	19.9 (9.9–31.8)	27.8 (19.7–37.3)	12.6 (7.9–21.5)	0.1246	NA	NA	NA
Median contrast volume(IQR), mL	120.0 (90.0–163.0)	117.5 (95.0–165.0)	120.0 (79.5–160.0)	0.7656	NA	NA	NA

Abbreviations: BA—brachial artery; FA—femoral artery; IQR—interquartile range; NA—not assessed; ND—not determined; RA—radial artery. Categorical outcomes: chi-squared test with simulated *p*-value (10^7^ replicates); post hoc test: Fisher’s exact test adjustment according to Benjamini–Hochberg method. For ordered larger contingency tables, the ordered approximative general independence test (10^7^ resamples) was used. Continuous outcomes: approximative Kruskal–Wallis’s test with 10^7^ resamples; post hoc test: Dunn’s test and the Holm adjustment; alpha = 0.05; reject H_0_ if *p* ≤ alpha/2. * Statistically significant.

**Table 4:** Perioperative and long-term complications.

Perioperative Complications	RA Group (*n* = 17)	BA Group (*n* = 9)	FA Group (*n* = 58)	All Patients (*n* = 84)
Access site complications	*n* (%)	*n* (%)	*n* (%)	*n* (%)
**Major**	0 (0)	1 (11.1)	7 (12.1)	8 (9.5)
Occlusion	0 (0)	0 (0)	0 (0)	0 (0)
Haematoma	0 (0)	0 (0)	4 (6.9)	4 (4.8)
Bleeding	0 (0)	1 (11.1)	1 (1.7)	2 (2.4)
Pseudoaneurysm	0 (0)	0 (0)	2 (3.4)	2 (2.4)
**Minor**	0 (0)	2 (22.2)	11 (18.9)	13 (15.5)
Occlusion	0 (0)	0 (0)	0 (0)	0 (0)
Haematoma	0 (0)	2 (22.2)	11 (18.9)	13 (15.5)
Bleeding	0 (0)	0 (0)	0 (0)	0 (0)
Summary	0 (0)	3 (33.3)	18 (31.0)	21 (25)
**MAE at 12 months**	***n*** **(%)**	***n*** **(%)**	***n*** **(%)**	***n*** **(%)**
Death	3 (17.6)	4 (44.4)	5 (8.6)	12 (14.3)
Major amputation	4 (23.5)	2 (22.2)	13 (22.4)	19 (22.6)
Re-PTA or bypass (TLR or TVR)	2 (11.8)	1 (11.1)	12 (20.7)	15 (17.8)
Myocardial infarction	0 (0)	0 (0)	2 (3.4)	2 (2.4)
Stroke	2 (11.8)	1 (11.1)	5 (3.5)	8 (9.5)
**Summary (all events)**	11 (64.7)	8 (88.9)	37 (63.8)	56 (66.7)
**Summary (patients with events)**	7 (41.2)	6 (66.7)	29 (50)	42 (50)
**MALE at 12 months**	***n*** **(%)**	***n*** **(%)**	***n*** **(%)**	***n*** **(%)**
Major amputation	4 (23.5)	2 (22.2)	13 (22.4)	19 (22.6)
Re-PTA or bypass (TLR or TVR)	2 (11.8)	1 (11.1)	12 (20.7)	15 (17.8)
Repeated ALI	2 (11.8)	2 (22.2)	7 (12.1)	11 (13.1)
**Summary (all events)**	8 (47.1)	5 (55.6)	32 (55.2)	45 (53.6)
**Summary (patients with events)**	6 (35.3)	3 (33.3)	25 (43.1)	34 (40.5)

Abbreviations: ALI—acute lower limb ischaemia; BA—brachial artery; FA—femoral artery; MAE—major adverse event; MALE—major adverse limb event; PTA—percutaneous transluminal angioplasty; RA—radial artery; TVR—target vessel revascularisation; TLR—target lesion revascularisation.

**Table 5:** Cox proportional hazards regression, major adverse events.

Covariate	b	SE	Wald	P	HR	95% CI of HR
Access site = ‘Brachial’	0.019559	0.55671	0.0012343	0.9720	1.01975	0.34246–3.03650
Access site = ‘Radial’	−1.31657	0.65040	4.09757	* 0.0429	0.26805	0.07492–0.95908
Rutherford stage = ‘IIB’	1.29269	0.42676	9.17549	* 0.0025	3.64257	1.57814–8.40757
Target vessel = ‘AA’	0.61379	1.10952	0.30603	0.5801	1.84742	0.20995–16.25564
Target vessel = ‘BTK’	−0.32042	1.06561	0.090418	0.7636	0.72584	0.08990–5.86011
Target vessel = ‘CFA’	0.25333	0.75989	0.11114	0.7388	1.28831	0.29053–5.71286
Target vessel = ‘CIA’	0.60224	0.68160	0.78068	0.3769	1.82620	0.48013–6.94610
Target vessel = ‘EIA’	3.31489	1.15999	8.16636	* 0.0043	27.51928	2.83291–267.32563
Target vessel = ‘Graft’	0.18943	0.48137	0.15486	0.6939	1.20856	0.47045–3.10472
Target vessel = ‘PA’	0.32409	0.52773	0.37715	0.5391	1.38277	0.49152–3.89013
Additional procedure = ‘No’	0.38637	0.33879	1.30061	0.2541	1.47162	0.75756–2.85876
Clinical success = ‘No’	2.04401	0.57854	12.48263	* 0.0004	7.72154	2.48453–23.99742
Diabetes mellitus = ‘Yes’	0.78023	0.39279	3.94574	* 0.0470	2.18197	1.01042–4.71190

Abbreviations: AA—abdominal aorta; BTK—below the knee; CFA—common femoral artery; CI—confidence interval; CIA—common iliac artery; EIA—external iliac artery; HR—hazard ratio; PA—popliteal artery; SE—standard error. * Statistically significant.

**Table 6:** Cox proportional hazards regression, major adverse limb events.

Covariate	b	SE	Wald	P	HR	95% CI of HR
Access site = ‘Brachial’	−1.02310	0.62711	2.66162	0.1028	0.35948	0.10516–1.22880
Access site = ‘Radial’	−1.16539	0.64254	3.28957	0.0697	0.31180	0.08850–1.09855
Rutherford stage = ‘IIB’	1.45839	0.39420	13.68746	* 0.0002	4.29902	1.98529–9.30926
Target vessel = ‘AA’	1.27547	1.06307	1.43952	0.2302	3.58040	0.44568–28.76313
Target vessel = ‘BTK’	−0.64437	1.05394	0.37381	0.5409	0.52499	0.06653–4.14268
Target vessel = ‘CFA’	0.85106	0.71158	1.43045	0.2317	2.34213	0.58063–9.44764
Target vessel = ‘CIA’	0.86280	0.59913	2.07387	0.1498	2.36979	0.73235–7.66830
Target vessel = ‘EIA’	3.44271	1.12245	9.40739	* 0.0022	31.27162	3.46500–282.22593
Target vessel = ‘Graft’	0.34019	0.48305	0.49597	0.4813	1.40521	0.54521–3.62178
Target vessel = ‘PA’	−0.0017039	0.51949	0.000010758	0.9974	0.99830	0.36063–2.76347
Additional procedure = ‘No’	0.38822	0.34302	1.28090	0.2577	1.47436	0.75269–2.88796
Clinical success = ‘No’	0.71874	0.52552	1.87052	0.1714	2.05184	0.73251–5.74745
Diabetes mellitus = ‘Yes’	0.26969	0.39667	0.46223	0.4966	1.30955	0.60183–2.84954

Abbreviations: AA—abdominal aorta; BTK—below the knee; CFA—common femoral artery; CI—confidence interval; CIA—common iliac artery; EIA—external iliac artery; HR—hazard ratio; PA—popliteal artery; SE—standard error. * Statistically significant.

## Data Availability

Data are contained within the article.

## Abbreviations

AHA	American Heart Association
ACC	American College of Cardiology
ALI	acute limb ischemia
BA	brachial artery
CDT	catheter-directed thrombolysis
CI	confidence interval
FA	femoral artery
HR	hazard ratio
MAE	major adverse event
MALE	major adverse limb event
MT	mechanical thrombectomy
PTA	percutaneous transluminal angioplasty
RA	radial artery
STILE	Surgery or Thrombolysis in Lower Extremity Ischemia
TOPAS	Thrombolysis Or Peripheral Artery Surgery
